# Salivary Gland Uptake on ^18^F‐Florbetaben PET Did Not Demonstrate Additional Diagnostic Value for Alzheimer's Disease

**DOI:** 10.1002/brb3.71199

**Published:** 2026-01-07

**Authors:** Hyun Woo Kwon, Saim Jung, Cheolmin Shin, Jong Hun Kim, Moon Ho Park

**Affiliations:** ^1^ Department of Nuclear Medicine Korea University Ansan Hospital Ansan South Korea; ^2^ Department of Psychiatry Korea University Ansan Hospital Ansan South Korea; ^3^ Department of Neurology Korea University Ansan Hospital Ansan South Korea

**Keywords:** Alzheimer's disease, PET, salivary gland

## Abstract

**Objectives:**

Saliva, like other body fluids, has been investigated as a source of biomarkers for Alzheimer's disease (AD) due to its accessibility. Recently, decreased amyloid‐PET uptake in the salivary glands of patients with AD was reported, prompting interest in the potential clinical relevance of that finding. This study evaluates the association between salivary gland uptake on ^1^ ^8^F‐florbetaben PET and cortical amyloid burden and assesses its diagnostic value.

**Methods:**

We retrospectively analyzed 73 patients who underwent ^18^F‐florbetaben PET imaging. Salivary gland uptake (SUVR_SG_) was measured and compared with the cortical amyloid burden, severity of dementia, and clinical diagnoses (cognitively normal, mild cognitive impairment, and dementia). Diagnostic performance was assessed using receiver operating characteristic (ROC) curve analyses.

**Results:**

The mean SUVR_SG_ was 1.43 ± 0.29, with no significant differences based on the cortical amyloid burden, severity of dementia, or clinical diagnosis. SUVR_SG_ did not correlate with the cortical amyloid burden (ρ = −0.103, p > 0.05). The area under the ROC curve and volume under the ROC surface indicated that SUVR_SG_ had poor diagnostic performance.

**Conclusions:**

Salivary gland uptake on ^18^F‐florbetaben PET was not associated with the cortical amyloid burden or clinical stage of AD, and it did not demonstrate diagnostic value. Further research is needed to explore its biological significance and standardization methods.

## Graphical abstract

Salivary gland uptake on ^1^ ^8^F‐florbetaben PET was not associated with cortical amyloid burden in patients across the cognitive spectrum. No significant differences in salivary gland amyloid‐PET uptake were observed according to dementia severity or clinical diagnosis. These findings suggest that salivary gland amyloid‐PET uptake has limited clinical utility for assessing Alzheimer's disease.

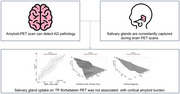

## 1 Introduction

Alzheimer's disease (AD), the most common cause of dementia, is characterized by the deposition of extracellular amyloid‐beta (Aβ) neuritic plaques and the accumulation of intracellular hyperphosphorylated tau protein in the brain (McKhann et al. 2011; McKhann et al. 1984). In the past, in vivo detection of these hallmark pathologies was not feasible, prompting extensive efforts to identify surrogate biomarkers outside the central nervous system. Such approaches have included histopathological detection of Aβ deposits in peripheral organs, including skin, subcutaneous tissue, intestine, and retina (Joachim et al. 1989; Hart et al. 2016); the identification of amyloid precursor protein in non‐neural tissues such as the myocardium, adrenal glands, bone marrow, and megakaryocytes (Arai et al. 1991); and measuring Aβ or tau levels in body fluids such as blood (Olsson et al. 2016; Sarto et al. 2023), saliva (Reale et al. 2020; Guo et al. 2025; Nazir 2024; Wolgin et al. 2022), urine (Wongta et al. 2020), nasal discharge (Jung et al. 2022), or tears (Gijs et al. 2021; van de Sande et al. 2023). However, those peripheral biomarkers have shown inconsistent results and are often limited by suboptimal sensitivity, specificity, or reproducibility (Rajendran and Krishnan 2024).

Recent advances in biomarker technology, particularly positron emission tomography (PET) imaging and cerebrospinal fluid (CSF) analysis, have enabled in vivo detection of AD pathology and are now established tools in the diagnostic workup of patients with AD, and their use is anticipated to increase with the introduction of new disease‐modifying therapies (McKhann et al. 2011; Leuzy et al. 2025). Amyloid‐PET imaging uses radiolabeled tracers that allow visualization of regional cortical Aβ deposition, which was previously restricted to postmortem histopathological examinations (Nordberg 2004).

Amyloid‐PET tracers bind selectively to cortical Aβ deposition, but they also exhibit non‐specifically elevated uptake throughout the white matter, regardless of AD pathology (Rabinovici et al. 2025). Similar uptake has also been visualized in extra‐cerebral structures such as the scalp, muscle, spleen, thyroid glands, salivary glands, and kidneys (D'Estanque et al. 2017; Neuraceq 2025; Kim et al. 2025).

Among those extra‐cerebral structures, the salivary glands are consistently captured within the field of view during brain PET scans, making them particularly amenable to incidental analysis without additional scanning protocols (Neuraceq 2025; Kim et al. 2025). Intriguingly, a recent study reported that salivary amyloid‐PET uptake was associated with cortical Aβ deposition (Kim et al. 2025). Given that the salivary glands have been associated with the secretion of potential biomarkers for AD (Reale et al. 2020; Guo et al. 2025; Nazir 2024; Wolgin et al. 2022) and the feasibility of including them in amyloid‐PET imaging (Neuraceq 2025; Kim et al. 2025), this study evaluates the clinical relevance of salivary gland amyloid‐PET uptake in patients with AD. Specifically, we evaluate the diagnostic value of salivary gland amyloid‐PET uptake in patients with AD.

## 2 Materials and Methods

### 2.1 Study Participants

We conducted a cross‐sectional retrospective study at a single medical center. More specifically, we reviewed the medical records of patients with suspected cognitive impairment who were referred for diagnosis to the Memory Clinic of Korea University Ansan Hospital from March 2023 to May 2025. All patients underwent ^18^F‐florbetaben brain PET‐CT imaging and a clinical diagnostic protocol that included routine blood tests, neuropsychological testing, and brain MRI, primarily to exclude the possibility of medical conditions and structural brain lesions that might influence cognitive function, such as acute stroke, brain tumors, hydrocephalus, and traumatic brain injury.

The patients with dementia fulfilled the criteria of probable AD proposed by the National Institute of Neurological and Communicative Disorders and Stroke and the Alzheimer's Disease and Related Disorders Association (McKhann et al. 1984). We used Petersen's criteria to diagnose mild cognitive impairment (MCI) (Petersen et al. 1999). Cognitively normal (CN) participants had normal performance (above −1.5 SD of norm) in all the tested domains of a neuropsychological test battery. In this study, the severity of dementia was evaluated using the global deterioration scale (GDS), with higher scores representing greater cognitive impairment (Reisberg et al. 1982).

In a sub‐classification of AD status performed using amyloid‐PET, the participants were classified as CN without amyloid deposition [CN group, CN(−)], MCI with amyloid deposition [MCI due to AD; MCI(+)], MCI without amyloid deposition [MCI initial syndromic diagnostic categories; MCI(−)], dementia with amyloid deposition [dementia due to AD; Dementia(+)], and dementia without amyloid deposition [dementia with non‐AD, Dementia(−)].

This study was approved by the Institutional Review Board of Korea University Ansan Hospital (approval no. 2025AS0154). Informed consent was not required because the study was retrospective.

### 2.2 PET Image Acquisition

All PET images were acquired using Discovery MI PET/CT scanners (GE Healthcare, Milwaukee, WI, USA), and the radiotracer ^18^F‐florbetaben (Neuraceq) was produced by DuChemBio (Seoul, South Korea). Brain amyloid‐PET images were acquired over the course of 20 min in the dynamic mode (4 × 5 min frames). Scanning was performed 90 to 110 min after the injection of a bolus (mean dose, 300 MBq, 8.1 mCi) into an antecubital vein. Non‐contrast‐enhanced CT (120 kVp, 50 mA, 3.75 mm thickness) was performed before the amyloid‐PET scan for attenuation correction. The scanning range was set from the vertex of the skull to the lower medulla oblongata. All amyloid‐PET images were reconstructed by a 3D‐ordered iterative algorithm (VPFX with 4 iterations with 34 subsets, quantification with Q.Clear; matrix size 256 × 256).

### 2.3 Image Analysis

Images were analyzed by one board‐certified staff nuclear medicine physician at Korea University Ansan Hospital, who was blinded to clinical data and used a commercially available workstation (Advanced Workstation version 4.8, GE Healthcare, Milwaukee, WI, USA). We analyzed the ^18^F‐florbetaben brain PET data by visualization and quantification. Visual interpretation was conducted by comparing the activity in the cortical gray matter with activity in the adjacent cortical white matter. In general, four regions of interest (the frontal, temporal, and parietal cortices and the posterior cingulate/precuneus) were interpreted with a visual rating of amyloid‐PET findings. Each of these brain regions was scored according to the regional cortical tracer uptake (RCTU) and brain amyloid plaque load (BAPL) systems (Neuraceq 2025; Sabri et al. 2015). The RCTU scoring system grades the tracer uptake in each of the above four regions (1 = no binding, 2 = minor binding, and 3 = pronounced binding), and those scores are converted into a single three‐grade scoring system for each PET scan. The BAPL scores are condensed into a binary interpretation [score of 1 classified as Aβ negative (Aβ^−^), and scores of 2 and 3classified as Aβ positive (Aβ^+^)].

For the quantification analyses of cortical amyloid burden, we used the centiloid scales (CL) of the brain (CL_B_) across seven subregions (global, frontal, posterior cingulate‐precuneus, lateral temporal, parietal, medical temporal, occipital, basal ganglia) (Klunk et al. 2015). For this analysis, data were evaluated using BTXBrain (Brightonix Imaging Ltd., Seoul, South Korea) within each brain region based on an automated anatomical labeling template (Kim et al. 2022). Values less than 10 CL robustly exclude neuritic amyloid load [CL_B_(−)], and values greater than 30 CL robustly identify the presence of brain neuritic amyloid plaques [CL_B_(+)]. The range between 10 and 30 CL has been suggested to capture an intermediate range, transitioning from sparse to moderate neuritic plaques [CL_B_(±)] (Iaccarino et al. 2025).

For the salivary glands, a sphere with a volume of 5 cm^3^ was drawn on each parotid gland. The white matter of cerebellum was used as a reference tissue, a sphere with a diameter of 5 cm^3^ was drawn on each cerebellar lobe, and the left and right average values were used as reference tissue uptake values. The average uptake ratio of the salivary glands (SUVR_SG_) was calculated by dividing the average uptake value of the parotid glands by that of the cerebellum.

### 2.4 Statistical Analyses

Descriptive data are presented as frequencies and percentages for categorical variables and means ± standard deviations or medians and interquartile ranges for continuous variables. Categorical variables were evaluated with chi‐square tests to assess difference between proportions, and continuous variables were evaluated with Student's *t*‐test or one‐way ANOVA (Mann‐Whitney *U* test or Kruskal‐Wallis *H* test, if the distribution was not normal), as appropriate. Bonferroni correction was used for *post‐hoc* comparisons. The relationships between different PET values and clinical characteristics were analyzed using Spearman's rank correlation coefficients. Additionally, a partial correlation analysis was performed to explore those relationships while adjusting for demographic and clinical characteristics that had statistically different distributions according to amyloid status.

A receiver operating characteristic (ROC) analysis of the area under the curve (AUC) was used to assess the diagnostic value of SUVR_SG_ for a dichotomic classification of amyloid‐PET scans (Aβ^+^ vs. Aβ^−^). Supplementary three‐dimensional ROC analyses produced the volume under a surface (VUS) to address the performance of SUVR_SG_ in making three simultaneous classifications by CL_B_ [CL_B_(−), CL_B_(±), and CL_B_(+)] and clinical diagnosis (CN, MCI, and dementia). A perfect diagnosis would be AUC = 1.0 and VUS = 1.0, and a noninformative test equivalent to chance would obtain an AUC value of 0.5 for the ROC curve and a VUS value of 0.167 (= 1/6) for the ROC surface. Based on a rough classifying system, AUC can be interpreted as follows: 0.9–1.0 = excellent; 0.8–0.9 = good; 0.7–0.8 = fair; 0.6–0.7 = poor; 0.5–0.6 = fail (Safari et al. 2016), and VUS can be interpreted as having discriminating power when both the VUS and its 95% CIs are above 0.167 (Xiong et al. 2006).

Statistical significance was declared when the two‐tailed *p* value was <0.05. SPSS (version 20.0; IBM SPSS, Chicago, IL, USA) and R (version 4.4.3; R Foundation for Statistical Computing, Vienna, Austria) were used for all the statistical analyses.

## 3 Results

### 3.1 Demographic Characteristics

We enrolled 73 participants in this study. The demographic and clinical characteristics of the participants are summarized in Table 1. The average age was 69.82 ± 10.01 years, and 34.2% of participants were male. Age, sex, and education did not differ between the dichotomic classifications of the amyloid‐PET scans (Aβ^−^ group *vs*. Aβ^+^ group) (*P* >0.05). However, the ApoE ε4 carrier was more prevalent in the Aβ^+^ group than in the Aβ^−^ group. The GDS scores and continuous CL_B_ scale were higher in the Aβ^+^ group than in the Aβ^−^ group (*P* <0.05). Also, the distributions of clinical diagnosis and the nominal CL_B_ scale differed between the Aβ^+^ and Aβ^−^ groups (*P* <0.05).

**TABLE 1:** Demographic and clinical characteristics.

	Total	Aβ^−^	Aβ^+^	*P*
	(n = 73)	(n = 35)	(n = 38)	
Age, years	69.82 ± 10.01	68.66 ± 11.14	70.89 ± 8.86	0.344
Sex, male	25 (34.2%)	15 (42.9%)	10 (26.3%)	0.149
Education, year	9.00 [6.00–12.00]	9.00 [6.00–12.00]	9.00 [6.00–14.00]	0.983
ApoE ε4 carrier^*^	21 (39.6%)	6 (20.7%)	15 (62.5%)	0.002
GDS	4.00 [3.00–5.00]	3.00 [3.00–4.00]	4.00 [3.00–5.00]	0.001
Clinical diagnosis				0.020
CN	3 (4.1%)	3 (8.6%)	0 (0.0%)	(0.392)
MCI	32 (43.8%)	19 (54.3%)	13 (34.2%)	(0.505)
Dementia	38 (52.1%)	13 (37.1%)	25 (65.8%)	(0.086)
CL_B_				
Continuous scale	52.31 [−4.14–91.00]	−4.69 [−12.00–5.00]	88.59 [63.44–103.52]	<0.001
Nominal scale				<0.001
CL_B_(−), <10	29 (39.7%)	29 (82.9%)	0 (0.0%)	(<0.001)
CL_B_(±), 10–30	4 (5.5%)	2 (5.7%)	2 (5.3%)	(1.000)
CL_B_(+), >30	40 (54.8%)	4 (11.4%)	36 (94.7%)	(<0.001)
SUVR_SG_	1.43 ± 0.29	1.44 ± 0.27	1.41 ± 0.31	0.654

Abbreviations: CN, cognitively normal; CL_B_, centiloid scale of the brain; GDS, global deterioration scale; MCI, mild cognitive impairment; MMSE, Mini‐Mental State Examination; SUVR_SG_, standardized uptake value ratio of the salivary glands.

Data are expressed as the mean ± SD, median [interquartile range], or actual counts (percentage), depending on their type and distribution.

Some data were missing, so the number of available data points for the ApoE ε4 allele was 53.

*P* values with parentheses were calculated from a pairwise *z*‐test with a Bonferroni correction to account for multiple testing and were used to determine the significance of the contribution for each subgroup of variables.

Using the binary interpretation of ^18^F‐florbetaben brain PET, Aβ^−^ indicates a BAPL score of 1, and Aβ^+^ indicates BAPL scores of 2 and 3.

### 3.2 Salivary Gland Uptake on Amyloid PET

**FIGURE 1:**
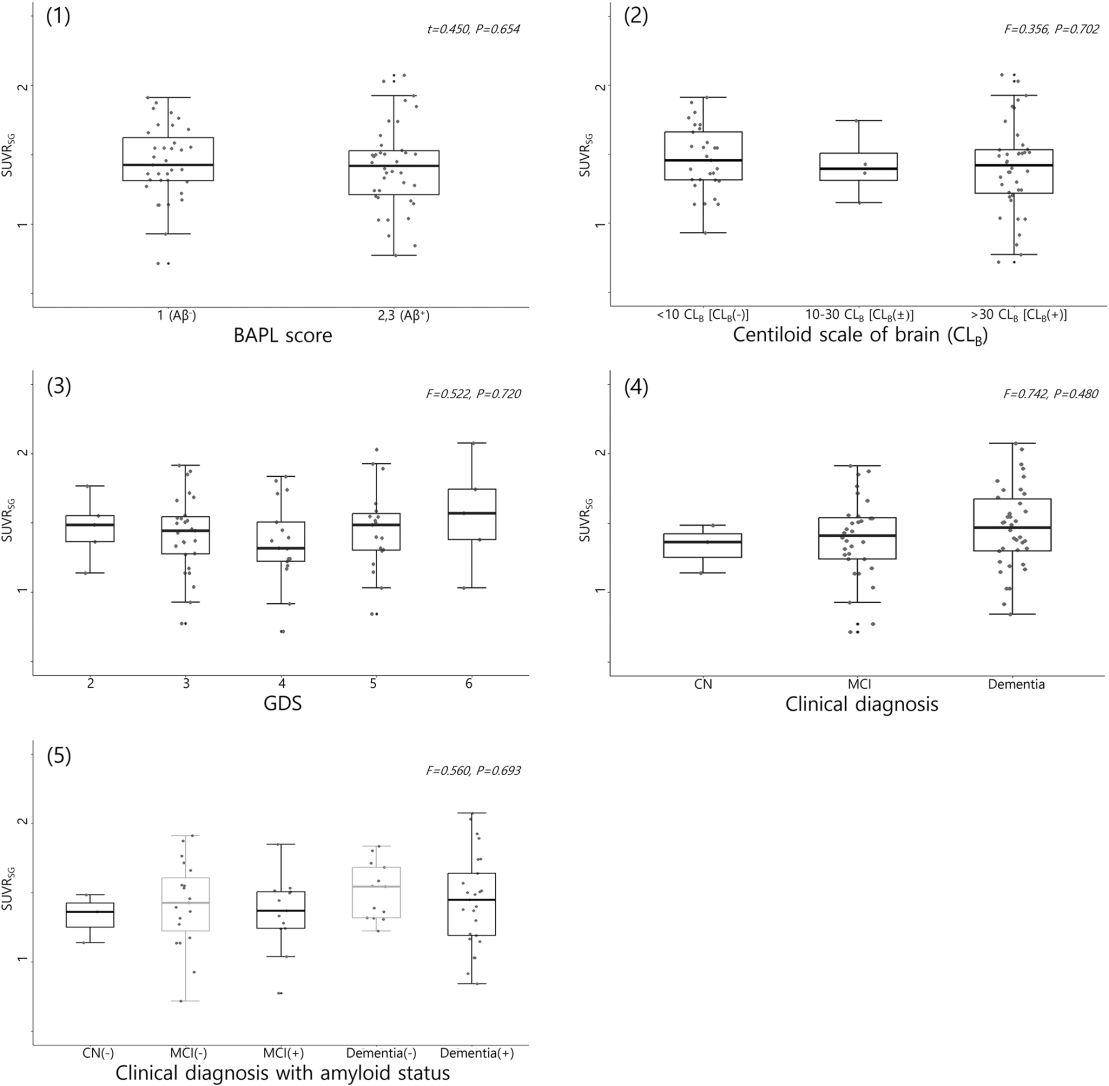
**Boxplots for SUVR_SG_**. Boxplots according to the BAPL score (1), CL_B_ (2), GDS (3), clinical diagnosis (4), and clinical diagnosis with amyloid status (5). The statistical parameters and *P* values, which were estimated using *t*‐tests or one‐way analysis of variance, are expressed in italics. The amyloid status is expressed with positive (+) or negative (−) symbols as a dichotomic classification of the BAPL scores from the amyloid‐PET scan results (Aβ^+^ vs. Aβ^−^). SUVR_SG_, standardized uptake value ratio of the salivary glands; BAPL, brain amyloid plaque load; CL_B_, centiloid scale of the brain; GDS, global deterioration scale; CN, cognitively normal; MCI, mild cognitive impairment.

The average SUVR_SG_ was 1.43 ± 0.29. Although the SUVR_SG_ was lower in the Aβ^+^ group (1.41 ± 0.31) than in the Aβ^−^ group (1.44 ± 0.27), the difference between the groups was not statistically significant (Table 1). Furthermore, SUVR_SG_ did not vary with the BAPL score, CL_B_, GDS, clinical diagnosis, or clinical diagnosis with amyloid status (Figure 1).

**FIGURE 2:**
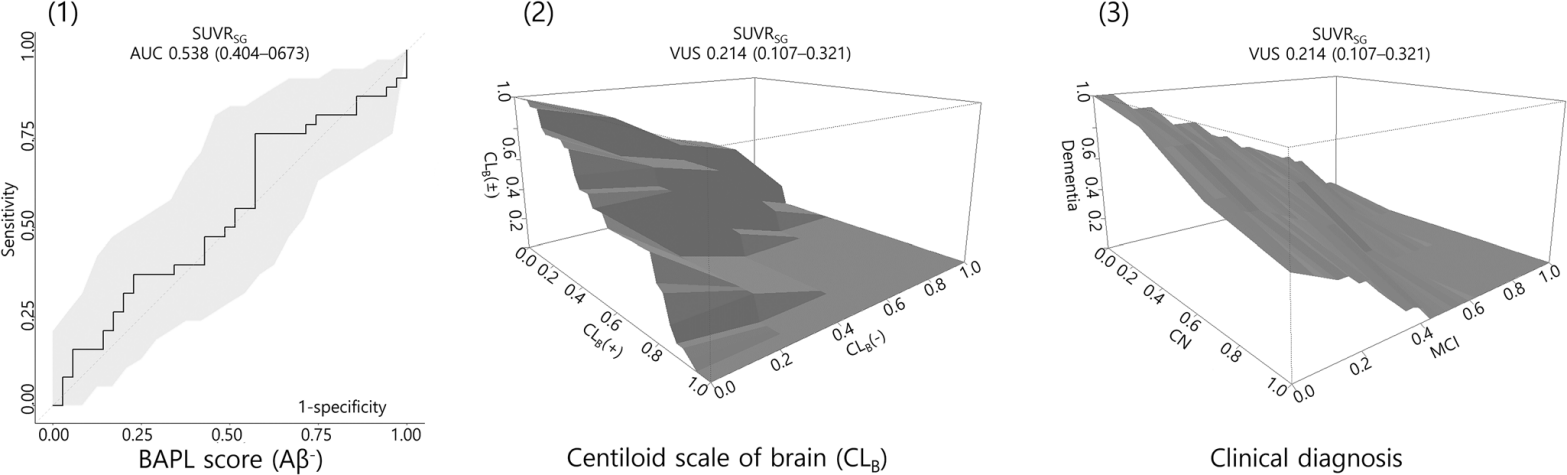
**Diagnostic value of SUVR_SG_ for the BAPL score, CL_B_, and clinical diagnosis**. Two‐ and three‐dimensional receiver operating characteristic curves or surfaces expressed according to the BAPL score (1), CL_B_ (2), and clinical diagnosis (3). Values are expressed as the AUC or VUS (95% confidence intervals). SUVR_SG_, standardized uptake value ratio of the salivary glands; AUC, area under the curve; BAPL, brain amyloid plaque load; VUS, volume under the surface; CL_B_, centiloid scale of the brain; CN, cognitively normal; MCI, mild cognitive impairment.

The diagnostic properties of SUVR_SG_ are summarized in Figure 2. When discriminating the Aβ^+^ group from the Aβ^−^ group, SUVR_SG_ was interpreted to have failed (<0.6 AUC). When discriminating among three diagnostic categories concurrently (i.e., CL_B_ [CL_B_(‐), CL_B_(±), and CL_B_(+)]) and clinical diagnosis (CN, MCI, and dementia), the VUS and 95% CIs of SUVR_SG_ were not above the chance level (<0.167 VUS).

**FIGURE 3:**
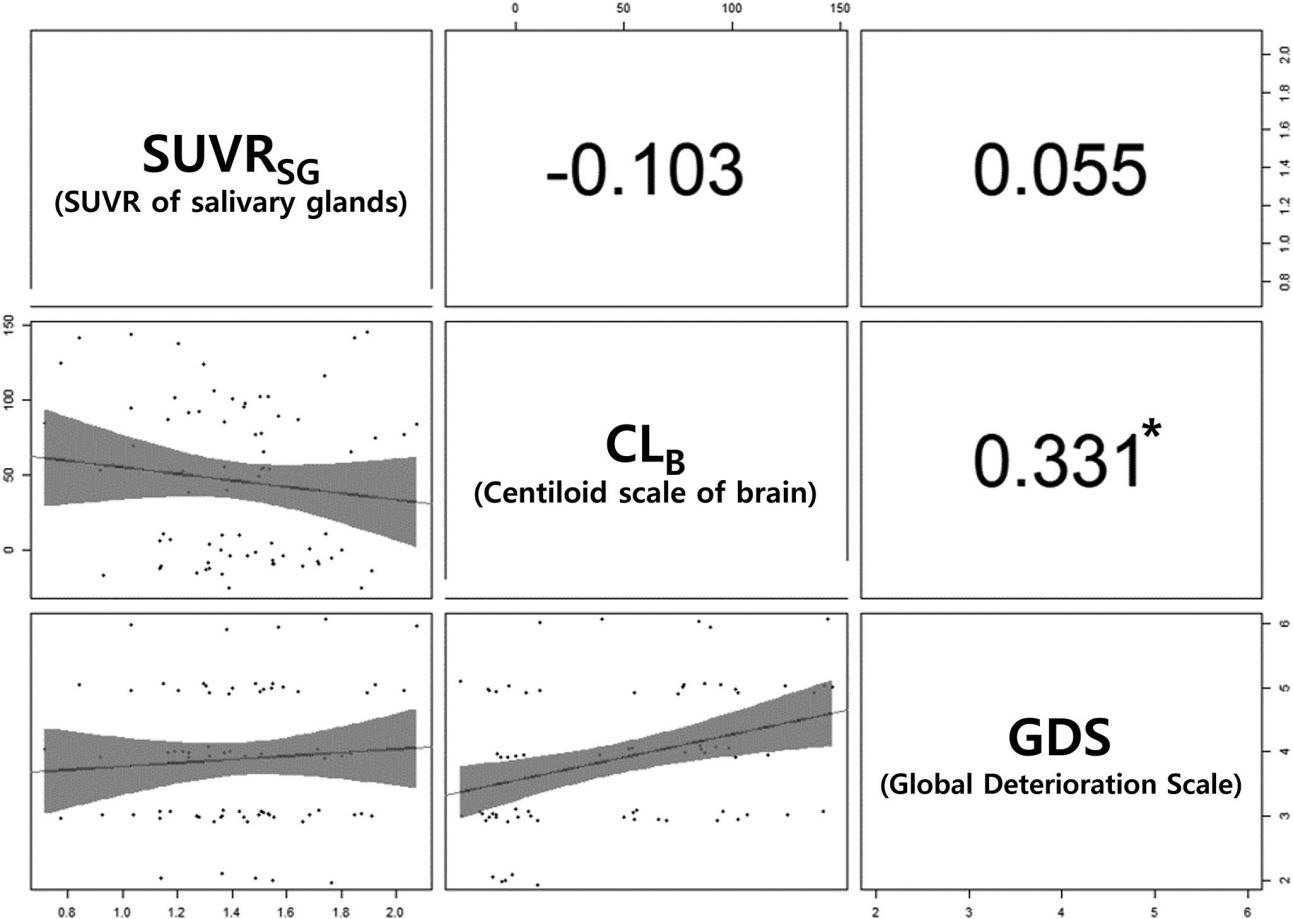
**Multivariable correlation matrix and scatterplots for SUVR_SG_, CL_B_, and GDS**. Values indicate Spearman's rank correlation coefficient. Correlation values with an asterisk (*) were statistically significant (*P* < 0.05). SUVR_SG_, standardized uptake value ratio of the salivary glands; CL_B_, centiloid scale of the brain; GDS, global deterioration scale.

The correlation analysis is summarized in Figure 3. The Spearman's rank correlation analysis demonstrated significant associations between CL_B_ and GDS (*ρ* = 0.331, *p* = 0.004). SUVR_SG_ correlated negatively with CL_B_, but without statistical significance (*ρ* = ‐0.103, *p* = 0.386). In the partial correlation controlling for the ApoE ε4 carrier, CL_B_ and GDS continued to have a statistically significant correlation (partial ρ = 0.209, *p* = 0.037). However, partial correlations between SUVR_SG_ and CL_B_ while controlling for the ApoE ε4 carrier still had no statistical significance (partial *ρ* = ‐0.103, *p* = 0.386).

## 4 Discussion

We used ^18^F‐florbetaben PET imaging to investigate the salivary gland amyloid‐PET uptake in patients with AD to evaluate its diagnostic value. In this study, salivary gland amyloid‐PET uptake did not correlate significantly with the cortical amyloid burden. Salivary gland amyloid‐PET uptake was also not associated with the severity of dementia or the clinical diagnosis (CN, MCI, or dementia). Overall, salivary gland amyloid‐PET uptake did not demonstrate additional diagnostic value for assessing cortical amyloid deposition in this study.

The association between the salivary glands and AD has been widely investigated because the salivary glands and saliva are potential sources of readily available biomarkers that can easily be acquired with low risk to the patient (Reale et al. 2020; Guo et al. 2025; Nazir 2024; Wolgin et al. 2022). Saliva might reasonably be expected to contain potential biomarkers for AD because the pathogenic molecules associated with AD might travel back and forth from the brain to the saliva via the blood–brain barrier, the blood–CSF barrier, the arachnoid villi, and perineural spaces (Pfaffe et al. 2011; Liu et al. 2023). Several pathophysiological links between the salivary glands and AD have been suggested, including local Aβ production by the ductal epithelium (Floden et al. 2020), reduced salivary gland secretion and mastication dysfunction secondary to impaired cranial nerve function in patients with AD (Femminella et al. 2014; Zalewska et al. 2021), and disturbance of the salivary microbiome in patients with AD (Sedghi et al. 2021). Nevertheless, investigations into salivary Aβ concentrations in AD patients have yielded inconsistent results, ranging from low (Tvarijonaviciute et al. 2020) or undetectable (Marksteiner et al. 2022) to elevated levels (Lee et al. 2017; Sabbagh et al. 2018). Furthermore, because definitive AD pathology—Aβ neuritic plaques and hyperphosphorylated tau—is restricted to the brain and cannot be definitely identified in peripheral tissues, evaluations of the relationship between the salivary glands and AD pathology remain inherently limited (Hyman et al. 2012).

In this study, we used ^18^F‐florbetaben as an amyloid‐PET tracer. This tracer, like other amyloid‐PET tracers, specifically binds to the Aβ‐pleated‐sheet structure, and PET imaging with this radiotracer has been shown to accurately detect Aβ neuritic plaques in the cortices of patients with AD (Neuraceq 2025; Villemagne et al. 2011). However, amyloid‐PET exhibits nonspecific tracer retention throughout cerebral white matter, regardless of the presence or absence of cortical Aβ uptake (Rabinovici et al. 2025). This signal in cerebral white matter does not reflect Aβ deposition. Although the precise mechanism remains unclear, it has been hypothesized that the tracers nonspecifically bind to beta‐sheets in the myelin basic protein (Stankoff et al. 2011).

In addition to intra‐cerebral uptake, some extra‐cerebral amyloid uptake is often visible during amyloid‐PET imaging. Physiologic uptake within the urinary tract (kidneys, renal pelvis, ureters, and bladder) and the enterohepatic circulatory system (liver, gallbladder, bile ducts, and small intestine) reflects the clearance and excretion pathways of the amyloid tracers (O'Keefe et al. 2009; Scheinin et al. 2007); however, those structures are typically outside the routine field of view during brain PET imaging. Conversely, salivary glands are routinely captured during standard brain scans due to their anatomical proximity to the brain, and they consistently exhibit elevated tracer uptake. Although the exact reason for the elevated amyloid‐PET uptake in salivary glands remains unclear, possible explanations include accumulation of the tracer or its radioactive metabolites, residual blood radioactivity (Neuraceq 2025), or tracer binding associated with adipose‐enriched tissue specific to the salivary glands that involves an undefined adipose tissue–Aβ interaction mechanism (Kim et al. 2025).

In this study, we investigated not only the association between salivary gland amyloid‐PET uptake and the cortical amyloid burden but also its diagnostic value for AD. Our ROC curve analyses show that salivary gland amyloid‐PET uptake failed to demonstrate adequate diagnostic accuracy in distinguishing the cortical amyloid burden. Additionally, three‐dimensional ROC analyses, which concurrently evaluated cortical amyloid burden categories and clinical diagnosis, yielded performance no better than chance. These findings indicate that salivary gland amyloid‐PET uptake is likely insufficient as an AD biomarker, especially compared with established methods such as amyloid‐PET imaging or CSF biomarkers.

A few previous studies investigated whether salivary gland amyloid‐PET uptake is associated with AD pathology. Although one study reported a negative correlation between salivary gland amyloid‐PET uptake and cortical amyloid burden (Kim et al. 2025), we found no statistically significant association. However, the previous study measured cortical amyloid burden using SUVR rather than the CL and reported a weak correlation (r = −0.188) that explained only 3.5% of the variance; it further reported associations between adiposity and both cortical and salivary gland amyloid uptake (Kim et al. 2025). We did not include BMI as a variable, which could partly account for the discrepancies between the results of the two studies. Additionally, methodological differences, such as variations in patient populations, sample size, and quantification methods, might also contribute to the differing results. Despite the inconsistent findings, even if salivary Aβ measurements reflect Aβ levels in CSF or blood, it is presumed that amyloid‐PET tracers, which specifically bind to the Aβ‐pleated‐sheet structure, are unlikely to indicate salivary gland amyloid‐PET uptake associated with the cortical amyloid burden. If any significant association exists, further research is required to determine whether known patterns in the relationship between CSF Aβ and cortical amyloid‐PET—such as the strong floor effect observed within the Aβ‐positive range (Salvado et al. 2019) or the interpretation of CSF Aβ as a state marker indicating the presence rather than the severity of neuritic plaques (Blennow and Hampel 2003)—also apply to salivary gland amyloid‐PET uptake. Further research is required to clarify the precise role of salivary gland amyloid uptake in relation to cortical amyloid burden and to validate its clinical relevance as a biomarker for AD.

This study is not without limitations. First, the small sample size raises the possibility of selection bias, and the lack of detailed information on MCI subtypes and dementia etiologies further restricts the clinical interpretation of our findings. As a result, the generalizability of our results may be limited, underscoring the need for larger, multi‐center studies to validate these observations. In addition, comprehensive cognitive profiles—including neuropsychological test scores or domain‐specific assessments—were not available for this analysis. Standardized methods for assessing SUVR_SG_ are also lacking; notably, the mean SUVR_SG_ observed here (1.43) was lower than that reported previously (2.16) (Kim et al. 2025), which may reflect methodological variability. Moreover, we did not include other core AD biomarkers, such as tau or CSF measures, nor did we incorporate metabolic indicators that have been associated with both cortical and salivary tracer uptake in prior studies (Gomez et al. 2018; Hida et al. 2012). The absence of these variables limits comparability with earlier findings and may have contributed to the null associations observed. Finally, neither salivary anatomical nor volumetric characteristics—such as gland size or adipose content—nor histopathological features of the salivary glands, nor potential confounding factors such as comorbidities or medications that may influence salivary gland physiology, were assessed. Future studies should integrate metabolic profiling as well as structural, volumetric, and histopathological evaluations to establish the clinical relevance of salivary gland PET measures in AD.

## 5 Conclusion

Salivary gland amyloid‐PET uptake was not significantly correlated with cortical amyloid burden. To our knowledge, this is the first study to examine the diagnostic value of salivary gland amyloid‐PET uptake for assessing the cortical amyloid burden, and the results suggest that it is insufficient for this purpose.

## Author Contributions

**Hyun Woo Kwon**: conceptualization, methodology, data curation, software, investigation, validation, formal analysis, and writing – original draft. **Saim Jung**: investigation, supervision, resources, and writing – original draft. **Cheolmin Shin**: investigation, supervision, resources, and writing – original draft. **Jong Hun Kim**: investigation, supervision, and resources. **Moon Ho Park**: investigation, validation, conceptualization, methodology, data curation, supervision, formal analysis, project administration, resources, writing – review & editing, writing – original draft, and visualization.

## Funding

The authors have nothing to report.

## Ethics Statement

This study was approved by the Institutional Review Board of Korea University Ansan Hospital (approval no. 2025AS0154). Informed consent was not required because the study was retrospective. This study was conducted in accordance with the Declaration of Helsinki.

## Conflicts of Interest

The authors declare no conflicts of interest.

## Data Availability

The data that support the findings of this study are available on request from the corresponding author.
